# Depth-dependent distribution patterns of ammonia- and nitrite-oxidizing microorganisms in the water column of stratified lakes

**DOI:** 10.1038/s41598-025-26324-2

**Published:** 2025-11-23

**Authors:** Albin Alfreider, Thomas Posch, Monika Summerer

**Affiliations:** 1 Department of Ecology, University of Innsbruck, 6020 Innsbruck, Austria; 2 Limnological Station, Department of Plant and Microbial Biology, University of Zurich, Kilchberg, Switzerland

**Keywords:** Alpine lakes, Freshwater lakes, Nitrification, Ammonia oxidation, Anammox, Ecology, Ecology, Environmental sciences, Microbiology

## Abstract

This study investigates the occurrence and diversity of ammonia- and nitrite-oxidizing prokaryotes in the water columns of four stratified lakes by analyzing 16S rRNA genes in DNA and RNA extracts. The taxonomic composition of nitrifiers differed between two lake types, with considerable depth-related variation within each lake. In large and deep lakes (Zürichsee and Achensee), the oxygenated hypolimnion was dominated by *Ca*. Nitrosopumilus, accounting for a quarter of the total 16S rRNA genes. *Nitrospira* rRNA genes were generally an order of magnitude less abundant than those of *Ca*. Nitrosopumilus. However, analyses of rRNA in the same samples revealed a significantly higher relative abundance of *Nitrospira* ribosomes, reaching up to one-third of the abundance of *Ca*. Nitrosopumilus ribosomes. *Nitrosospira* and *Ca*. Nitrotoga were the main nitrifiers in the upper hypolimnion of the smaller lakes Piburger See and Hechtsee. The anoxic, deep hypolimnion of these lakes was dominated by the anaerobic ammonium-oxidizing bacterium *Ca*. Anammoximicrobium. The vertical distribution of *Ca*. Anammoximicrobium was further resolved using droplet digital PCR, revealing a strong correlation with ammonium concentrations. These findings provide new insights into the niche preferences of nitrifiers and anammox bacteria in lakes, highlighting the importance of depth-dependent environmental gradients in shaping their distribution.

**Supplementary Information:**

The online version contains supplementary material available at 10.1038/s41598-025-26324-2.

## Introduction

The nitrification process is carried out by organisms capable of utilizing reduced inorganic nitrogen compounds as an energy source while simultaneously fixing inorganic carbon to fulfill their carbon requirements^1,2^. For more than 100 years, nitrification was traditionally viewed as a two-step process catalyzed by two distinct microbial groups: ammonia oxidizing (AOB) and nitrite-oxidizing bacteria (NOB). Recent advances have significantly altered our understanding of the nitrification process and renewed the interest in studying the ecology of nitrifiers in both engineered and natural habitats. In particular, the discovery of ammonia-oxidizing archaea (AOA) as fundamental contributors to nitrification in many environments^3,4^ and, more recently, the encounter of bacteria that catalyze complete nitrification (“Comammox”)^5,6^ has greatly expanded our view of the function and diversity of microbes in the nitrification process. In addition to the aerobic oxidation performed by nitrifiers, anaerobic ammonia oxidizing (anammox) bacteria (AMX) are also able to catalyze the oxidation of ammonium^7,8^. These bacteria use nitrite as an electron acceptor under anaerobic conditions. AMX are not only important in engineered biological nitrogen removal processes including wastewater treatment systems, but these bacteria have also been detected ubiquitously in anoxic environments, including aquatic systems^9,10^.

Nitrification research is often explored in engineered systems, and wastewater treatment plants in particular are frequently model systems for the study of niche preferences and environmental adaptations of nitrifying microbes^11,12^. In natural environments, most studies focus on soil habitats^13,14^. Investigations in aquatic environments are mainly carried out in marine systems^15,16^. The importance of the nitrification and the anammox process in freshwater systems and specifically the niche differentiation of AMX, AOA and/or AOB in the water column of lakes is less well understood. The lack of knowledge is not only due to the limited number of studies conducted in lakes. The diversity of ammonia-oxidizing organisms (AOOs) and NOB in lakes is strongly influenced by variations in climatic conditions, altitude, lake morphology and trophic status. This makes it difficult to predict which taxa are likely to dominate and to formulate a general concept for modelling these microorganisms in lakes. In general terms, however, Thaumarchaeota accompanied by *Nitrospirae* are often the most important ammonia oxidizers in the water column of deep and oligotrophic lakes and different representatives of AOB and NOB are often encountered in smaller and more productive lakes^17–27^. However, seasonality also plays a major role in the occurrence of different groups of taxa, as recently shown in a study in the oligotrophic Flathead lake by Peoples et al.^28^: While *Nitrosomonadaceae* (AOB) and *Ca.* Nitrotoga (NOB) dominate at depth in summer, *Nitrososphaerota* (AOA) and *Nitrospirota* (NOB) became more important in the winter season.

The objective of this study was to explore the vertical distribution of AOOs and NOB in the water column of two different lake types based on 16S rRNA sequence analysis.16S rRNA gene sequences (rDNA) were used to estimate abundance, whereas cDNA derived from ribosomal RNA (rRNA) was analyzed as a proxy for potential activity. The investigation strategy was driven by the hypothesis that the distribution of different AOOs and NOB is strongly depth dependent during stratification of the lakes. Sampling of different layers in the water column was performed to demonstrate whether pronounced oxygen and redox gradients are associated with the presence of different groups of AOB and NOB and to examine which environmental parameters affect niche specialization and the co-occurrence of nitrifying communities and AMX. Furthermore, the hypolimnion of one lake was sampled at high resolution to better resolve the vertical distribution of AMX using droplet digital PCR for quantification.

## Results and discussion

### Physico-chemical lake characteristics and overall microbial diversity

**Fig. 1:**
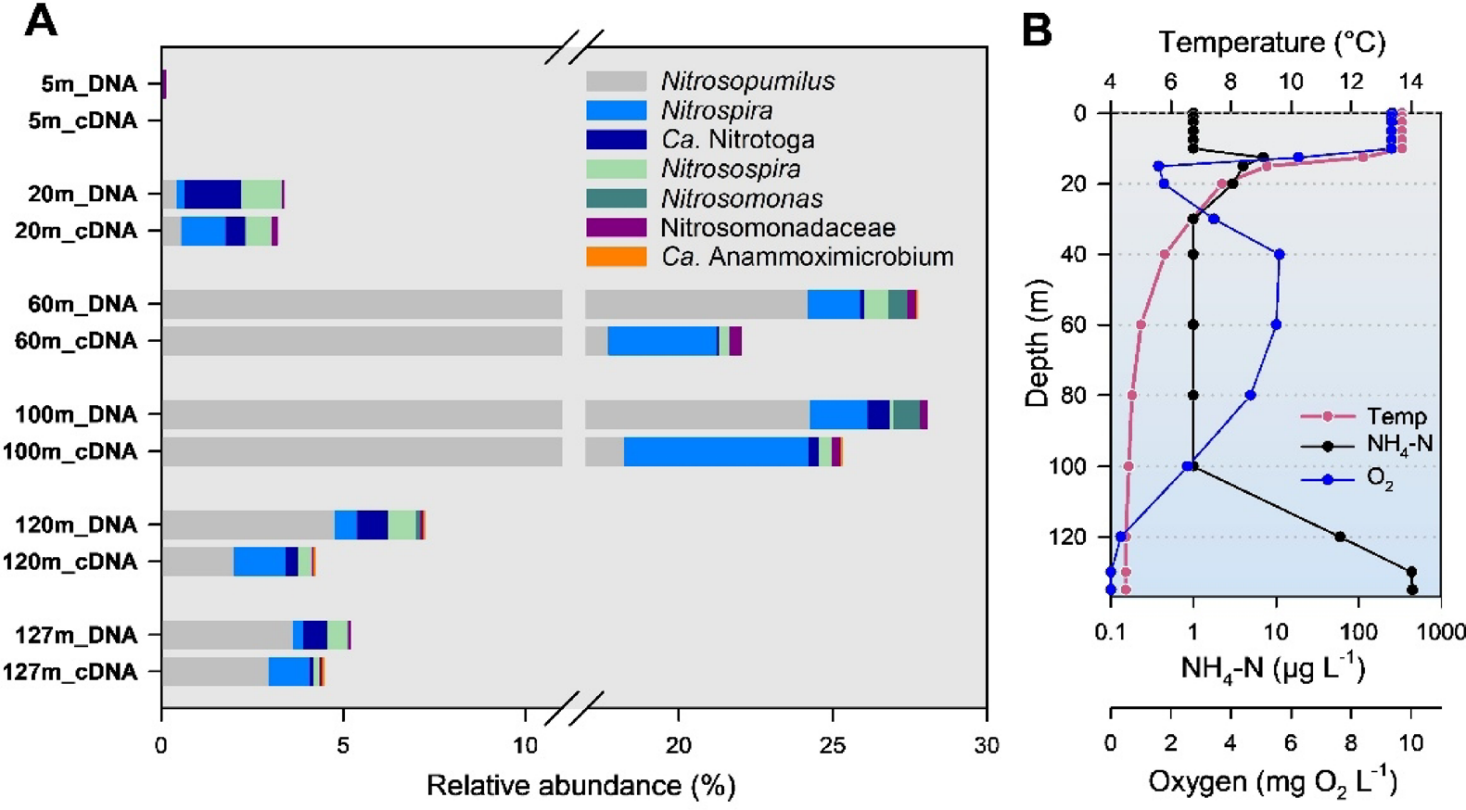
Vertical distribution and phylogenetic affiliation of nitrifiers in lake Zürichsee (ZUR), indicated as percentage of the total number of NGS-based 16S rDNA amplicon sequences based on DNA and cDNA data sets. “Nitrosomonadaceae” include genera other than *Nitrosomonas* and *Nitrosospira* (plot A). Taxa with a proportion of less than 0.1% of the total number of sequences are not shown. Water column profiles of ammonium, temperature and dissolved oxygen are shown in plot B.

At the time of sampling in November, lake Zürichsee (ZUR) exhibited a pronounced metalimnetic oxygen minimum between 15 and 20 m depth (Fig. 1). Dissolved oxygen (DO) levels increased again at depths below 20 m depth before dropping significantly below a depth of 100 m. The water column below 130 m was free of oxygen and the ammonium concentration reached 0.447 mg L^− 1^ NH_4_-N at 135 m depth near to the lake bottom. *Cyanobacteria* were the dominant group in the epilimnion of the lake (Supplementary Fig. S1), with *Planktothrix* being the largest group within this taxon. In addition to various representatives of *Proteobacteria* and *Planctomycetota*, *Nitrososphaeria* (*Ca*. Nitrosopumilus) was particularly abundant in the oxygen-rich hypolimnion (Supplementary Fig. S1). *Gammaprotoebacteria* are mainly found in the deep hypolimnion (120 and 127 m depth), with the genera *Methylobacter* and *Creonthrix* dominating the cDNA fraction.

**Fig. 2:**
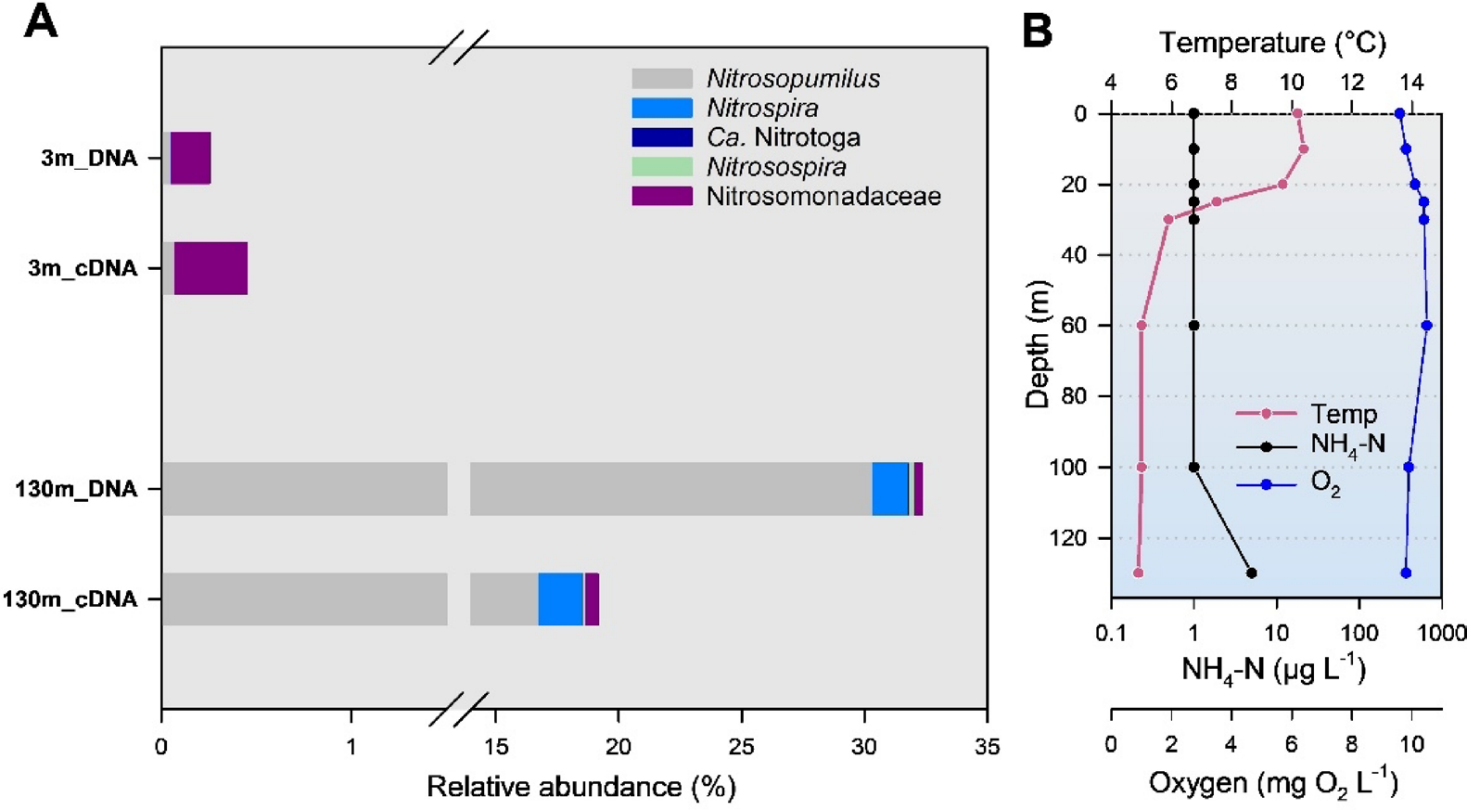
Vertical distribution and phylogenetic affiliation of nitrifiers in lake Achensee (ACH), indicated as percentage of the total number of NGS-based 16S rDNA amplicon sequences based on DNA and cDNA data sets. “*Nitrosomonadaceae*” include genera other than *Nitrosomonas* and *Nitrosospira* (plot A). Taxa with a proportion of less than 0.1% of the total number of sequences are not shown. Water column profiles of ammonium, temperature and dissolved oxygen are shown in plot B.

The DO - profile in lake Achensee (ACH) showed little depletion of oxygen throughout the entire water column during summer stratification (Fig. 2). Thus, the ultra-oligotrophic ACH was characterized by the absence or very low levels of reduced compounds, including ammonium, and very low nutrient concentrations. In comparison to the lake ZUR, the differences in microbial diversity in lake ACH are particularly pronounced in the epiliomnion. In this layer, *Alpha*- and *Gammaproteobacteria* dominate together with cyanobacteria, while *Bacteroidia* were also numerically abundant (Supplementary Fig. S2). In the hypolimnion, however, *Ca*. Nitrosopumilus is of similar quantitative importance as in lake ZUR. This taxon dominates this part of the pelagic zone, together with several representatives within *Proteobacteria*, *Planctomycetes* and *Verrucomicrobiae*.

**Fig. 3:**
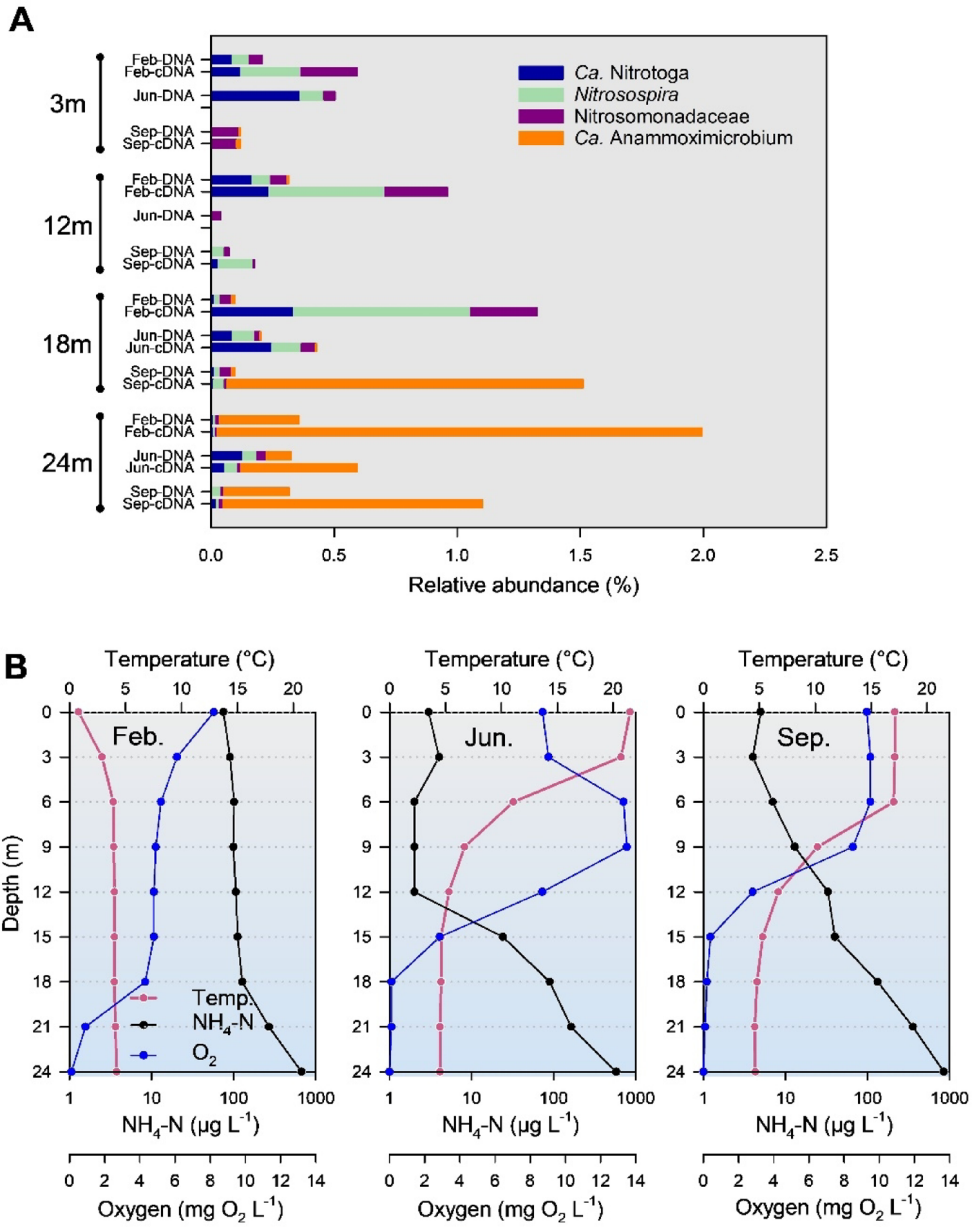
Vertical distribution and phylogenetic affiliation of nitrifiers in lake Piburger See (PIB), indicated as percentage of the total number of NGS-based 16S rDNA amplicon sequences based on DNA and cDNA data sets. “*Nitrosomonadaceae*” include genera other than *Nitrosomonas* and *Nitrosospira* (plot A). Results are based on DNA and cDNA data sets obtained in February, June and September. Taxa with a proportion of less than 0.1% of the total number of sequences are not shown. Water column profiles of ammonium, temperature and dissolved oxygen are shown in plot B.

Lake Piburgersee (PIB) showed a typical vertical summer stratification pattern at the sampling dates of late June and early September (Fig. 3). The temperature gradient, however, was more pronounced in September. DO was strongly depleted during summer stratification at depths below 18 m, associated with a strong accumulation of ammonium in the reducing environment of the deep hypolimnion (Fig. 3). Significant supersaturation of DO concentrations occurred in June at depths of 6 to 9 m. An inverse temperature stratification was established during winter under the ice cover at the sampling in late February. Accordingly, DO and ammonium gradients were less pronounced and only the sample taken at a depth of 24 m was below the detection limit (0.01 mg O₂ L⁻¹) for dissolved oxygen. In PIB, cyanobacteria are an important microbial group in the epilimnion and upper hypolimnion (Supplementary Fig. S3). High densities of cyanobacteria also occurred in February with reduced light intensity due to the ice cover. A broad distribution covering the entire water column and all sampling dates was observed for different representatives of *Gammaproteobacteria* and *Verrucomicrobiae*. *Alphaproteobacteria* were mainly found in the upper water layers, *Actinobacteria* and *Bacteroidia* are ubiquitous in the pelagic zone of PIB, with depth-dependent and seasonally varying abundances (Supplementary Fig. S3). At the oxygen-free sampling depth of 24 m, several taxa within the *Desulfobacterota* are numerically significant.

**Fig. 4:**
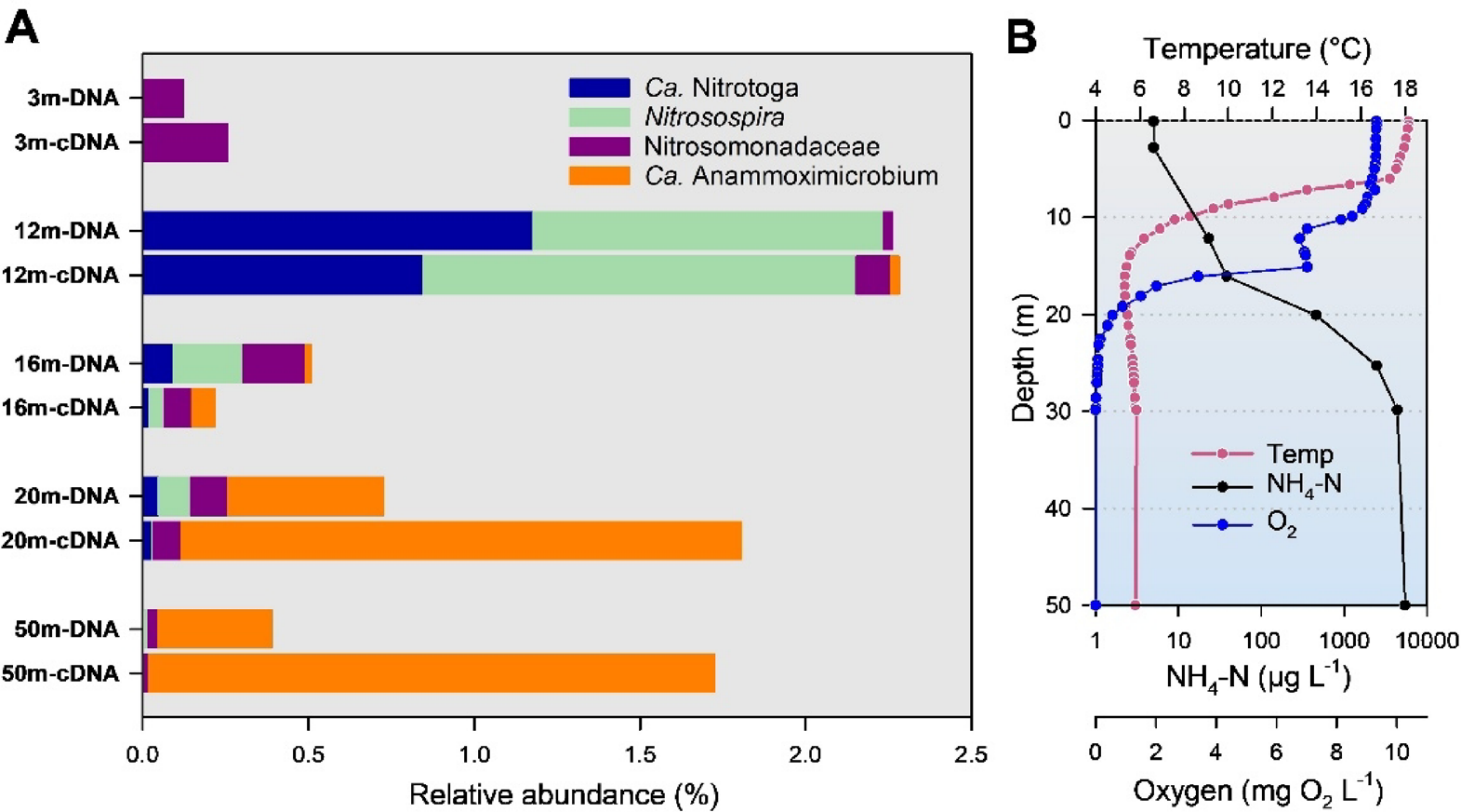
Vertical distribution and phylogenetic affiliation of nitrifiers in lake Hechtsee (HEC), indicated as percentage of the total number of NGS-based 16S rDNA amplicon sequences based on DNA and cDNA data sets. “*Nitrosomonadaceae*” include genera other than *Nitrosomonas* and *Nitrosospira* (plot A). Taxa with a proportion of less than 0.1% of the total number of sequences are not shown. Water column profiles of ammonium, temperature and dissolved oxygen are shown in plot B.

Lake Hechtsee (HEC) is meromictic and showed anoxic conditions from a depth of 20 m to the deepest sampling depth of 50 m at the time of sampling in September (Fig. 4). The hypolimnion was characterized by high ammonium concentrations with a maximum of 5.4 mg L^− 1^ NH_4_-N in 50 m depth (Fig. 4). The taxonomic composition of the microbial communities in lake HEC showed some similarities with PIB. Cyanobacteria were strongly represented in the epilimnion, accounting for more than half of the 16S rDNA sequences in the cDNA fraction at 3 m depth (Supplementary Fig. S4). Different groups of *Gammaproteobacteria* dominated the depths of 16 and 20 m, a region characterized by a sharp oxygen gradient. However, the diversity within the *Gammaproteobacteria* was high and differed significantly from the composition in PIB (data not shown). For example, purple sulphur bacteria related to the genera *Thiocystis* and *Thiodictyon*, were abundant at a depth of 20 m. These *Chromatiaceae* did not occur in PIB and are typical inhabitants of the chemocline of meromictic lakes^29^. HEC also harboured some archaeal representatives, including methanogens at the deepest sampling depth (50 m), which were not detected in the 16S rRNA libraries obtained from all other lakes (Supplementary Figs. S1-S4).

### Nitrifiers in deep lakes

In terms of relative abundances (percentage of 16S rRNA gene sequences), *Nitrososphaerota* (also known as *Thaumarchaeota*), affiliated with *Ca.* Nitrosopumilus was the dominant group of ammonia oxidizers in the oxygenated hypolimnion of deep lake ZUR, with relative abundances just over 24% of the total 16S rDNA sequences obtained at 60 and 100 m depth (Fig. 1). In the deep hypolimnion at depths of 120 and 127 m, a zone with oxygen concentrations below 1 mg O_2_ L^− 1^ (Fig. 1), the proportion of *Ca.* Nitrosopumilus was 20% of that in the oxic hypolimnion. The relative abundance of AOA in the epilimnion of lake ZUR was less than 1%. A similar pattern was observed in lake ACH. The proportion of *Ca.* Nitrosopumilus in the total prokaryotic rRNA sequences was 30% in the DNA and 17% in the RNA fraction, while AOA were almost absent in surface waters (relative abundances < 0,1%) (Fig. 2).

Other studies in freshwater systems support these observations: *Nitrososphaeria* are often the most important ammonia oxidizers in the water column of large and oligotrophic lakes^19,20,24,30^, with the recently described *Ca.* Nitrosopumilus limneticus as the dominant AOA in the hypolimnion of European perialpine lakes^27^. In line with our results, their distribution in the water column is often characterized by an increase in abundance with depth, with the highest numbers observed in the hypolimnion. Inhibition by light, competition for substrates with phototrophs and high grazing pressure may be responsible for the low numbers of AOA in lake surface waters^17–20,31,32^.

In contrast to AOOs, NOB are less well-characterized in lakes, and freshwater systems in general. Recent investigations suggest that the genus *Nitrospira* is the dominant guild of NOB in the water column of deep lakes^20,24,33^. These observations were also confirmed by our study, and *Nitrospira* was the most important group of NOB in terms of relative numbers in the well oxygenated hypolimnion of both lakes, ZUR and ACH (Figs. 1 and 2). All other taxa of AOB and NOB were always below 1% (relative abundances), with the exception of the water samples taken at a depth of 20 m from lake ZUR. Here, *Nitrosospira* (AOB) and *Ca*. Nitrotoga were the most important nitrifying representatives in the DNA fraction with relative abundances of 1.1 and 1.6% respectively.

Based on electron transfer and thermodynamics, the theoretical ratio of AOB to NOB in a stable nitrifying system should be 2:1^34^. Especially for wastewater treatment systems, variations of the NOB/AOB ratio are well described and reasons for the imbalance have been intensively investigated^35,36^. Cell activity is likely to be an important factor, and it was shown that the activity affects the growth balance of AOB and NOB and consequently the numerical ratio of AOB/NOB^36^. In natural systems, several studies reported the co-occurrence of AOA and nitrite-oxidizing *Nitrospira* in terrestrial systems^37^, but also in samples retrieved from lake sediments^38^. In the pelagic realm of several pre-alpine deep lakes, Alfreider et al.^20^ reported potential interactions between AOA and *Nitrospira*, as indicated by a significant correlation between archaeal *amoA* and *hcd* genes and the abundance of *aclA* genes (encoding ATP-citrate lyase in the rTCA cycle) in *Nitrospira*. However, *Nitrospira* was generally an order of magnitude less abundant than AO. Similar results were also reported by Herber et al.^24^ in a study conducted in the deep oligotrophic Lake Constance. They showed that *Nitrospiraceae*, consisting of members of the genus *Nitrospira*, closely followed those of the *Nitrosopumilaceae*, which were also most abundant in the hypolimnion. However, as in our study, AOA were generally an order of magnitude more abundant than *Nitrospira* OTUs. Interestingly, in marine systems, AOA usually outnumbered *Nitrospinae* (the dominant bacterial nitrite oxidizers in the open ocean) by a factor of 10. Kitzinger et al.^39^ showed that *Nitrospinae* have a four times higher biomass yield and five times higher growth rate than AOA under *in* *situ* conditions. Given that most molecular ecological studies rely on DNA-based analyses, they may not accurately reflect microbial activity. To better assess the potential activity of different taxa, we analyzed the ratio of cDNA (rRNA) to rDNA for AOA and Nitrospira as a proxy for relative activity. In Lake ZUR, this approach revealed a notable increase in the ratio of *Nitrospira* (based on cDNA) relative to Thaumarchaeota (Fig. 1). For example, in the oxic hypolimnion of lake ZUR, the AOA/NOB ratios derived from DNA extracts were 14.3:1 (60 m) and 12.9:1 (100 m depth). In the RNA (cDNA) fraction, however, the ratios were 4.9:1 (60 m) and 3.1:1 (100 m depth). A similar but less pronounced trend was also observed in Lake ACH (Fig. 2). Clearly, further research is needed to determine whether cell activity influences the AO and NOB ratios by using proxies other than 16S rRNA markers, including the determination of individual biovolumes and functional markers (e.g. *amoA*).

### Nitrifiers in small stratified lakes

Investigations carried out at lake PIB in February, June and September showed that aerobic AOOs and NOB were mainly related to the genera *Nitrosospira* and *Ca*. Nitrotoga (Fig. 3). However, their distribution did not show a clear depth-dependent pattern and within the group of AOB other bacteria from the family *Nitrosomonadaceae* occasionally dominated (Fig. 3). Relative abundances of nitrifying bacteria, based on rDNA sequences, were generally below 1% of the total prokaryotic sequences obtained per sample. The highest relative abundance occurred in February at a depth of 18 m in the cDNA fraction, with *Nitrosospira* representing 0.72% of the total 16S rRNA sequences. In contrast, no NOB affiliated with canonical *Nitrospira* were detected in this study, which is consistent with previous findings reporting the absence of Comammox *Nitrospira* in the water column of Lake PIB based on *amoA* gene quantification by ddPCR^40^. The 16S rDNA amplicon sequences retrieved from lake HEC were dominated by the same nitrifiers as observed in lake PIB: *Nitrosospira* and *Nitrotoga* are the main AOB and NOB in the epilimnion of the lake (Fig. 4), both bacterial groups peaked in the upper hypolimnion (12 m depth) of the lake.

**Fig. 5:**
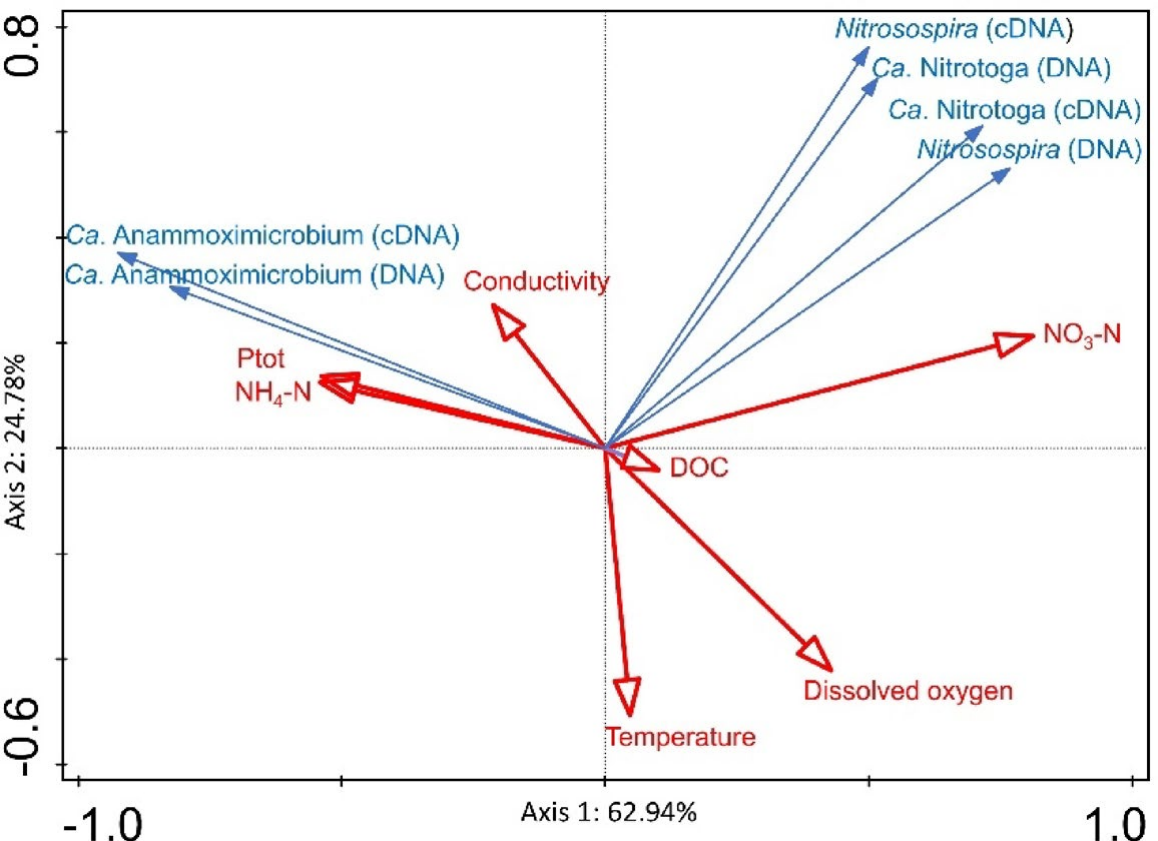
RDA biplot of relative abundances of *Nitrosospira*, *Ca*. Anammoximicrobium and *Ca*. Nitrotoga based on DNA and cDNA sequence analysis and selected environmental parameters obtained from lakes PIB and HEC.

Redundancy analysis (RDA) integrating temperature, dissolved oxygen, conductivity, ammonium, nitrate, total phosphorus, and dissolved organic carbon (data shown in Supplementary Table 1) confirmed that these environmental variables together explain most of the observed variation in the distribution of nitrifying and anammox taxa in lakes PIB and HEC (Fig. 5). Axis 1 (explaining 62.9% of the variance) primarily reflected the oxygen and nutrient gradient, separating the oxygenated, nitrate-rich upper water column from the anoxic, ammonium-enriched hypolimnion. *Ca*. Anammoximicrobium (DNA and cDNA) was strongly correlated with NH₄–N, total phosphorus, and conductivity, which supports its preference for oxygen-depleted, nutrient-rich conditions. *Nitrosospira* (AOB) and *Ca*. Nitrotoga (NOB) clustered towards the opposite side of the plot, aligned with dissolved oxygen and nitrate (NO₃–N), indicating dominance in suboxic to oxic upper hypolimnetic zones where nitrification proceeds aerobically. Their partial overlap suggests tight coupling between ammonia and nitrite oxidation processes.

Due to seasonal changes in light conditions, periodic patterns of mixing and stratification, and winter ice cover in northern hemisphere lakes, the amount and availability of different nitrogen forms are highly dynamic over an annual period ^41^. Although reduced oxygen availability and low temperatures during winter can limit nitrification, autumn mixing and the reduced light availability for phytoplankton caused by snow or ice cover can lead to increased ammonium concentrations and reduced competition for ammonium, resulting in higher nitrification rates^42^. Seasonal dynamics of limnological parameters strongly influence the activity and distribution of AOOs. In summer, low DO concentrations in the hypolimnion of small stratified lakes can inhibit ammonia oxidizers, especially when spatial separation of nitrate and ammonium occurs. Light availability is another critical factor influencing the ecology of freshwater nitrifiers^43^. AOOs are typically inhibited by light; however, recent studies have revealed adaptive strategies. Podowski et al.^25^ proposed that *Nitrosospira* species may utilize proteorhodopsin to supplement their energy metabolism under conditions of substrate limitation or when ammonia oxidation is photoinhibited, suggesting a mechanism enabling these organisms to persist in high-light environments. Furthermore, competition with cyanobacteria, as reported in studies from lakes Taihu and Okeechobee^44,45^ can suppress nitrification.

The distribution of AOA and AOB is also shaped by lake morphometry and related limnological characteristics. AOA are often rare in shallow lakes and in the upper water columns of large and deep lakes. In contrast to deep lakes, where Thaumarchaeota are often the most abundant nitrifiers in the oxygenated aphotic zone throughout the year, the hypolimnion of small stratified lakes is often characterized by low DO concentrations that overlap with inhibitory factors in the euphotic zone during summer^19–21^. AOB display high taxonomic heterogeneity, with representatives of *Nitrosomonas* and *Nitrosospira* frequently detected across diverse lake environments^20,23,25,26^. Notably, most studies investigating AOB and AOA distributions have focused on lake sediments rather than the water column^46,47^, highlighting a gap in our understanding of pelagic nitrification processes.

### Depth dependent distribution of *Ca*. Anammoximicrobium

Based on 16S rRNA gene (DNA) sequence analyses, *Ca*. Anammoximicrobium was identified as the dominant AMX in the deep hypolimnion of lakes PIB and HEC (Figs. 3 and 4). AMX were not detected, with the exception of a few sampling depths with very low numbers, in the water column of the large lakes ACH and ZUR (Figs. 1 and 2). Remarkably, based on the assumption that RNA: DNA ratios reflect the potential activity of a population, the ribosomal RNA: DNA ratios of *Ca*. Anammoximicrobium in PIB and HEC suggest an increased level of activity under oxygen-depleted conditions (Figs. 3 and 4), emphasizing the importance of the anammox process in the deep hypolimnion. Depth-dependent ammonium and DO concentrations were also determining factors for the distribution of *Ca*. Anammoximicrobium when relative sequence abundances were used for analyses (Fig. 5).

*Ca*. Anammoximicrobium belongs to a novel genus within the Planctomycetes and was originally discovered in a wastewater bioreactor^48^. Recent studies also indicated that these representatives are not only found in engineered systems, but are also important for the oxidation of ammonium in natural aquatic systems, including the hypolimnion of stratified lakes^49,50^. Despite this, the specific ecological requirements and niche preferences of *Ca*. Anammoximicrobium in natural systems remain poorly understood.

**Fig. 6:**
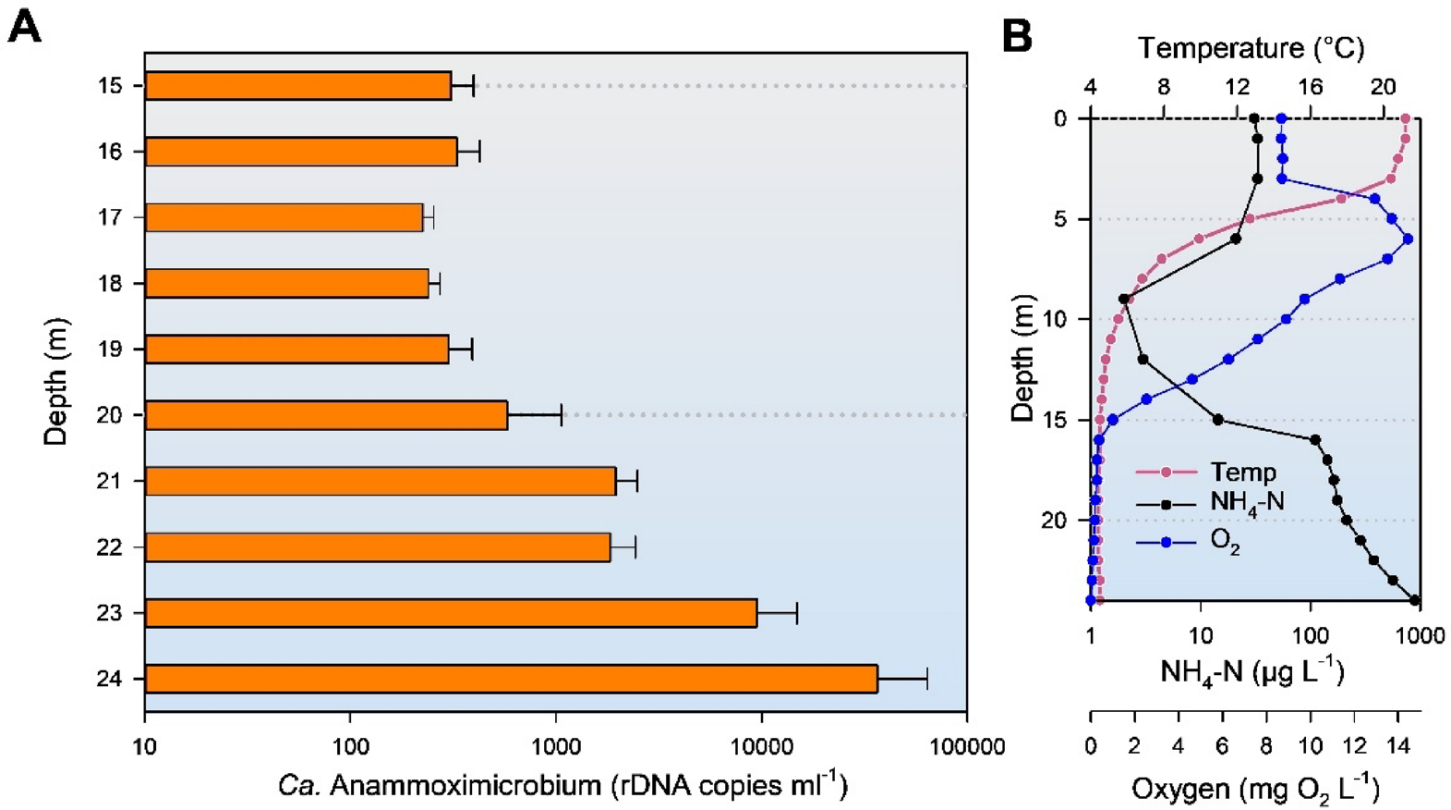
Vertical distribution of *Ca.* Anammoximicrobium rDNA gene copy numbers based on ddPCR analysis in the hypolimnion of lake PIB (plot A). Please note the logarithmic scale. Water column profiles of ammonium, temperature and dissolved oxygen are shown in plot B.

To better resolve the vertical distribution and potential niche preferences of AMX, the hypolimnion of PIB was sampled at one-metre intervals from a depth of 15 m downward during a separate sampling campaign. The abundance of AMX was quantified using ddPCR. *Ca*. Anammoximicrobium counts remained relatively stable between 15 and 20 m (Fig. 6). Below 21 m, its abundance increased markedly, reaching a maximum at 24 m close to the bottom of the lake. At this depth, the abundance of *Ca*. Anammoximicrobium was approximately two orders of magnitude higher than in the upper hypolimnion. On the day of microbial sampling (July 7, 2021), the hypolimnion showed stratification conditions typical of the summer period: the DO values decreased with increasing depth starting from 1 mg O_2_ L^− 1^ at 15 m depth. The deep hypolimnion was free of DO (Fig. 6). At the same time, there was a strong accumulation of ammonium in the reducing environment of the water column (Fig. 6). Spearman’s rank correlation analysis with key limnological parameters (ammonium, DO, temperature) showed a highly significant relationship of *Ca*. Anammoximicrobium abundance with DO (*r* = -0,81, *p* < 0.01) and ammonium concentrations (*r* = 0,81, *p* < 0.01) (Supplementary Table 2). This association has also been demonstrated using relative sequence abundances based on 16S rRNA gene (rDNA) or rRNA-derived cDNA reads assigned to *Ca*. Anammoximicrobium relative to the total prokaryotic reads (Fig. 5). No correlation was found between temperature and *Ca*. Anammoximicrobium. In the hypolimnion of lake PIB the temperature of the sampled water column ranged between 4 and 5 °C at this time of the year (Fig. 6). This narrow temperature range was likely insufficient to influence the distribution of *Ca*. Anammoximicrobium.

These results are consistent with the known physiology of AMX, which thrive in oxygen-depleted environments and rely on nitrite produced by aerobic ammonia oxidizers such as AOA and AOB. However, the same organisms (AOA, AOB and AMX) also compete for ammonia and it has been suggested that the affinity for ammonia plays an important role in the competition between AOA/AOB^51^ and their association with AMX^52^. As the anammox process is inhibited by oxygen, interactions between aerobic and anaerobic AOOs are most likely restricted to oxic/anoxic boundary regions in the water column of lakes. This has already been shown for marine systems in the stratified water column of the Black Sea, where the diffusion of nitrite generated by microaerophilic AOA and AOB present in the upper low-oxygen region supported AMX in the adjacent lower suboxic region^53^. The absence of AMX in the water column of deep lakes ACH and ZUR could be attributed to differences in stratification patterns, the oxygenated hypolimnion and nutrient availability, which do not provide the necessary environmental conditions for AMX to thrive.

## Conclusion

Through the combined analysis of the abundance of 16S rRNA genes (rDNA) and the relative abundance of ribosomal RNA (reflecting potential activity), supported by ddPCR results, it was demonstrated that the composition and vertical structuring of AOOs, NOB and AMX are strongly modulated by limnological gradients. In large, deep and oligotrophic lakes (ZUR and ACH), the oxygenated hypolimnion was consistently dominated by AOA affiliated with *Ca.* Nitrosopumilus, while *Nitrospira* represented the principal NOB, albeit at lower relative abundances. The higher cDNA: DNA ratios observed for *Nitrospira* may indicate a greater ribosomal content relative to AOA, suggesting a possible decoupling between population size and ribosome-based activity potential. However, this interpretation should be viewed cautiously, as differences in ribosome numbers and growth strategies among distinct taxa may influence these ratios independently of metabolic activity. In contrast, smaller lakes (PIB and HEC) exhibited a more heterogeneous assemblage of nitrifiers, dominated by AOB of the genus *Nitrosospira* and NOB affiliated with *Ca.* Nitrotoga, particularly colonizing the upper hypolimnion. Compared to ZUR and ACH, AOB and NOB generally showed lower abundances and less consistent depth patterns, which implies that the niche distribution of nitrifiers is subject to a complex or stochastic control in PIB and HEC. AMX, dominated by *Ca*. Anammoximicrobium, were exclusively detected in the anoxic hypolimnia of the smaller lakes. High-resolution vertical profiling combined with ddPCR quantification revealed a pronounced depth-dependent increase in AMX abundance, which was strongly correlated with decreasing dissolved oxygen and increasing ammonium concentrations. These findings suggest that *Ca*. Anammoximicrobium occupies a distinct ecological niche in stratified freshwater systems and plays a potentially significant role in nitrogen loss processes under anoxic conditions.

Our results highlight the influence of physicochemical stratification in shaping the composition and function of nitrifying and anammox microbial communities in freshwater lakes. Their depth-specific occurrence observed across different lake types underscore the importance of incorporating vertical and seasonal variability into models of microbial nitrogen cycling in lakes.

## Methods

### Study sites and sampling

Lake Piburger See (PIB) is a small, dimictic, oligo-mesotrophic lake located in a crystalline area of the Tyrolean Alps, Austria (Table 1). PIB is usually ice-covered from early December to April; following a brief spring overturn, the thermal stratification persists until the autumn overturn in November or December, with maximum surface temperatures of 20–25 °C in July and August. The physical and chemical properties of PIB have been described in detail in previous studies^20,54,55^. The second small lake Hechtsee (HEC), which is permanently meromictic and characterized by a pronounced chemocline and thermocline, has strictly anoxic conditions in the hypolimnion of the lake^19^. Accordingly, the hypolimnion is also characterized by high concentrations of phosphorus and ammonium. Mesotrophic Lake Zürichsee (ZUR) is a large and deep pre-alpine lake (Table 1). The lake is usually monomictic, with holomixis being the exception caused by lake warming. From May to October, a stable thermocline develops between 8 and 15 m depth and ZUR usually develops a distinct metalimnetic oxygen minimum. Dissolved oxygen concentrations usually decrease significantly below 100 m depth. During the stagnation period, the deep hypolimnion is also rich in ammonium. Achensee (ACH) is also a deep lake but has one tenth of the surface area compared with ZUR (Table 1). ACH is monomictic and oxygen-saturation is always above 80% throughout the water column. Accordingly, ACH has very low concentrations of reduced compounds. ACH also has very low nutrient levels, which is characteristic for the lake’s ultra-oligotrophic status.

**Table 1:** Main characteristics of the study sites Piburger see (PIB), Hechtsee (HEC), Achensee (ACH) and Zürichsee (ZUR). Lakes are sorted by increasing water volume.

Lake	Location	Altitude (m a.s.l.)	Area (km^2^)	Max depth (m)	Volume (10^6^ m^3^)	Mixing type	Trophic state
PIB	46° 19′ N, 10° 88′ E	913	0.134	24.6	1.82	dimictic	oligo-mesotrophic
HEC	47° 61′ N, 12° 16′ E	542	0.28	57	8.8	meromictic	mesotrophic
ACH	47° 27′ N, 11° 42′ E	929	6.80	133	454	monomictic	ultra-oligotrophic
ZUR	47° 25′ N, 8° 64′ E	406	66.82	136	3900	monomictic	mesotrophic

To assess the seasonal dynamics of dimictic lake PIB, samples for ribosomal 16S DNA- and cDNA-based sequence analysis were taken during winter stagnation (February), and twice during summer stagnation (July and September) from water depths of 3, 12, 18 and 24 m at the deepest part of the lake. All other lakes with meromictic or monomictic water column stratification patterns (Table 1) were sampled once. HEC samples were collected in September from five depths with an increased vertical sampling resolution at the thermocline/chemocline (sampling depths 3, 12, 16, 20, 50 m). Water samples in lake ZUR were obtained in November from 5, 20, 60, 100, 120 and 127 m depth, when the water column was still characterized by a stable thermal stratification (Fig. 1). ACH samples were collected only at two depths in the epilimnion (3 m) and the deep hypolimnion (130 m) in summer, as this lake is characterized by constant environmental conditions over the entire water column in the hypolimnion (Fig. 2). In July 2021, the hypolimnion of PIB was sampled again; at this time at one-metre intervals starting at 15 m depth, with three parallel samples at each sampling depth and six replicates at 24 m depth near the lake bottom. These water samples were used for the quantification of AMX.

All samples were collected using Schindler-Patalas sampling devices (UWITEC, Mondsee, Austria). For high-resolution sampling of *Ca*. Anammoximicrobium in lake PIB, we used a compact Schindler-Patalas sampler (20 cm in height). This sampling device minimizes disturbance during sample retrieval and was suitable for the 1 m vertical resolution applied to our lake profiles. Prior to sampling, depth profiles of temperature (resolution: 0.01 °C), dissolved oxygen (resolution and detection limit: 0.01 mg O₂ L⁻¹) and conductivity (resolution and detection limit: 1 µS cm⁻¹) were determined using a multiparameter probe (YSI model 6600; Yellow Springs Instruments, OH). In addition to probe measurements, dissolved oxygen concentrations were quantified at sampling depths using the Winkler titration method. Nutrient analyses were performed using standard colorimetric methods and ion chromatography (Dionex DX-120, Dionex Inc., Sunnyvale, USA). Dissolved organic carbon (DOC) was measured using a TOC/TN-Analyzer (TOC-5000, Shimadzu, Kyoto, Japan). Laboratory-established limits of detection (LOD) and/or limits of quantification (LOQ) were as follows: NH₄⁺–N: LOD 1.5–4.1 µg L⁻¹, LOQ 4.7–12.4 µg L⁻¹; NO₃⁻–N: LOD 31 µg L⁻¹ (smallest standard 22.6 µg L⁻¹); Ptot: LOD 1.3 µg L⁻¹, LOQ 3.8 µg L⁻¹; DOC: LOD 43.3 µg L⁻¹, LOQ 95.8 µg L⁻¹. The chemical analysis of the lake water obtained from ZUR was provided by the Zurich Water Supply.

Water samples for DNA and RNA analyses were processed in the field, with the objective of minimizing processing and handling time due to the short half-life times of RNA and its high variability in environmental samples^56^. Microbial communities were collected by filtering a maximum volume of lake water (between 800 and 1500 mL, depending on filter clogging caused by varying population densities) from each sampling depth onto polyethersulfone filters (pore size 0.22 μm, Millipore, Bedford, MA). The filters were immediately placed on dry ice, transported to the laboratory and stored at -80 °C until DNA and RNA extraction.

### Nucleic acid-extraction and 16S rRNA analysis

To avoid a bias caused by potential variations in lysis extraction and efficiency for DNA and RNA using different extraction methods, DNA and RNA were co-extracted from the same samples using a commercially available kit (PowerWater©RNA Isolation Kit, Qiagen Inc., USA) according to the manufacturer’s protocol. After cDNA synthesis from the RNA extracts (SuperScript™ III Reverse Transcriptase, Thermo Fisher Inc., USA) following the instructions of the manufacturer, the amount of DNA and cDNA was measured fluorometrically (Quantus^Tm^, QuantiFluor^®^dsDNA and QuantiFluor^®^ ssDNA System chemistry, Promega Corporation, USA).

Prokaryotic communities were assessed by sequencing the V4 region of the 16S rRNA gene using the primer pair 515f Modified (5-GTGYCAGCMGCCGCGGTAA-3) / 806r Modified (GGACTACNVGGGTWTCTAAT)^57^. Amplicon sequencing was performed using Illumina MiSeq sequencing technology (Microsynth AG, Switzerland). Raw data were demultiplexed, quality filtered and adaptor trimmed by the sequencing company. Read quality was checked using FastQC software, and untrimmed sequences were discarded using Cutadapt v1.9.2.dev0^58^. Merging of the trimmed forward and reverse reads of the paired-end reads, with a minimum overlap of 15 bases, was performed using the software USEARCH software version 8.1.1861^59^. All sequences were further processed using the amplicon analysis pipeline SILVAngs (SILVAngs 1.4)^60^. Briefly, using SILVAngs each sequence read was aligned using the SILVA Incremental Aligner against the SILVA SSU rRNA SEED and quality controlled^60^. Sequence reads of less than 50 aligned nucleotides and low-quality reads with more than 2% ambiguities, or 2% of homopolymers, were not included in further analysis. Putative contaminants and artefacts, as well as reads with a low alignment quality (50 alignment identity, 40 alignment score reported by SINA), were also excluded from further analysis. The remaining sequences were dereplicated and the unique reads were clustered into operational taxonomic units (OTUs) and taxonomically classified. The dereplication and clustering were performed using VSEARCH (version 2.15.1]^61^ with identity criteria of 1.00 and 0.98, respectively. Classification was performed with BLASTn (2.2.30+]^62^ using the default settings of the non-redundant version of the SILVA SSU Ref dataset (release 138.1; http://www.arb-silva.de) as the classification reference. The classification of each OTU reference read was mapped to all reads that were assigned to the respective OTU. This provides quantitative information (number of individual reads per taxonomic path), within the limitations of PCR and sequencing technique biases, as well as, multiple rRNA operons. Reads with no or weak classifications, where the function “(% sequence identity + % alignment coverage)/2” did not exceed the value of 93, remain unclassified. Relative abundances of AOOs and NOB are the ratios of the reads from a specific taxon to all reads in each sample (expressed as a percentage of the total number of prokaryotes present).

### Droplet digital PCR

Quantification of *Ca.* Anammoximicrobium abundances was accomplished using a droplet digital PCR (ddPCR) system (QX200™, Bio-Rad Laboratories, Hercules, USA), as previously described in Alfreider et al.^20,21^ and Harringer and Alfreider^40^. Droplet were generated using a robotic droplet generator (AutoDG™ Instrument, Bio-Rad). The ddPCR reactions were prepared using an EvaGreen Supermix (QX200; Bio-Rad) in 96-well plates according to the manufacturer’s instructions. Primer design and evaluation is described below. The optimal annealing temperatures and primer concentrations for ddPCR were determined through a series of temperature gradient experiments and the testing of different primer concentrations. Droplet signal measurements were performed using a droplet reader (QX200, Bio-Rad) and data were analysed using the QuantaSoft AP software (Bio-Rad). The fluorescence amplitudes of positive and negative droplets and the reliability of the automated threshold settings was evaluated by visual examination. Unlike real-time PCR, ddPCR provides absolute quantification without the need for calibration curves (ddPCR relies on mimicking limiting dilution and Poisson statistics) and eliminates the need for technical replicates. In addition, as an endpoint measurement, ddPCR allows nucleic acid quantification independent of the reaction efficiency, resulting in a positive-negative call for each droplet.

Several broad-range 16S rRNA primers previously developed for detecting AMX in various engineered systems (bioreactors, wastewater treatment plants) and different freshwater ecosystems^63–65^ were tested with lake PIB hypolimnion samples using ddPCR. However, these primers failed to generate specific amplicons from the water samples. Sequence analyses revealed that while the primers matched most known anammox representatives - including *Ca*. Scalindua, *Ca*. Brocadia, *Ca*. Anammoxyglobus, Kuenenia and *Ca*. Jettenia - they did not cover *Ca*. Anammoximicrobium. These primer pairs showed different numbers of mismatches across the 16S rRNA sequence of *Ca*. Anammoximicrobium. To overcome this limitation, we adapted an existing 16S rRNA primer pair set (Amx694F/Amx960R)^63,66^ to include *Ca.* Anammoximicrobium. Based on temperature gradient ddPCR experiments, the primer pair Amx694F_mod (5’-GRG GTG AGY GGA ACT GAT G-3’) and Amx960R_mod (5’-SCT CCA CCG CTT GTG YGA GC-3’) gave a clearly distinguishable fluorescence signal band and the lowest number of unspecified signals (rain) with DNA when using an annealing temperature of 62 °C. A primer concentration of 200 nM was determined to be optimal. This modified primer pair was subsequently used for all further ddPCR analyses of PIB hypolimnion water samples.

### Statistical analysis

Redundancy analysis (RDA) implemented in the software package CANOCO 5^67^ was used to test evaluate and visualize the influence of different environmental factors (temperature, dissolved oxygen, conductivity, ammonium, nitrate, total phosphorus and DOC) on the distribution of different AOOs and NOB in the lakes.

## Supplementary Information

Below is the link to the electronic supplementary material.

Supplementary Material 1

## Data Availability

The 16S rRNA gene sequences and associated metadata have been deposited in the Sequence Read Archive (SRA) of NCBI under the project numbers PRJNA1034802 (lake PIB) and PRJNA1092999 (lakes ACH, HEC, ZUR).
