# Supplementary material for: Depth-dependent distribution patterns of ammonia- and nitrite-oxidizing microorganisms in the water column of stratified lakes
Source: Sci Rep. 2025 Nov 23;15:42232. doi: 10.1038/s41598-025-26324-2 (PMC12658065; doi:10.1038/s41598-025-26324-2)
Supplement: Supplementary file 1 — Supplementary Material 1 [file 41598_2025_26324_MOESM1_ESM.pdf]

Supplementary material Alfreider et al.

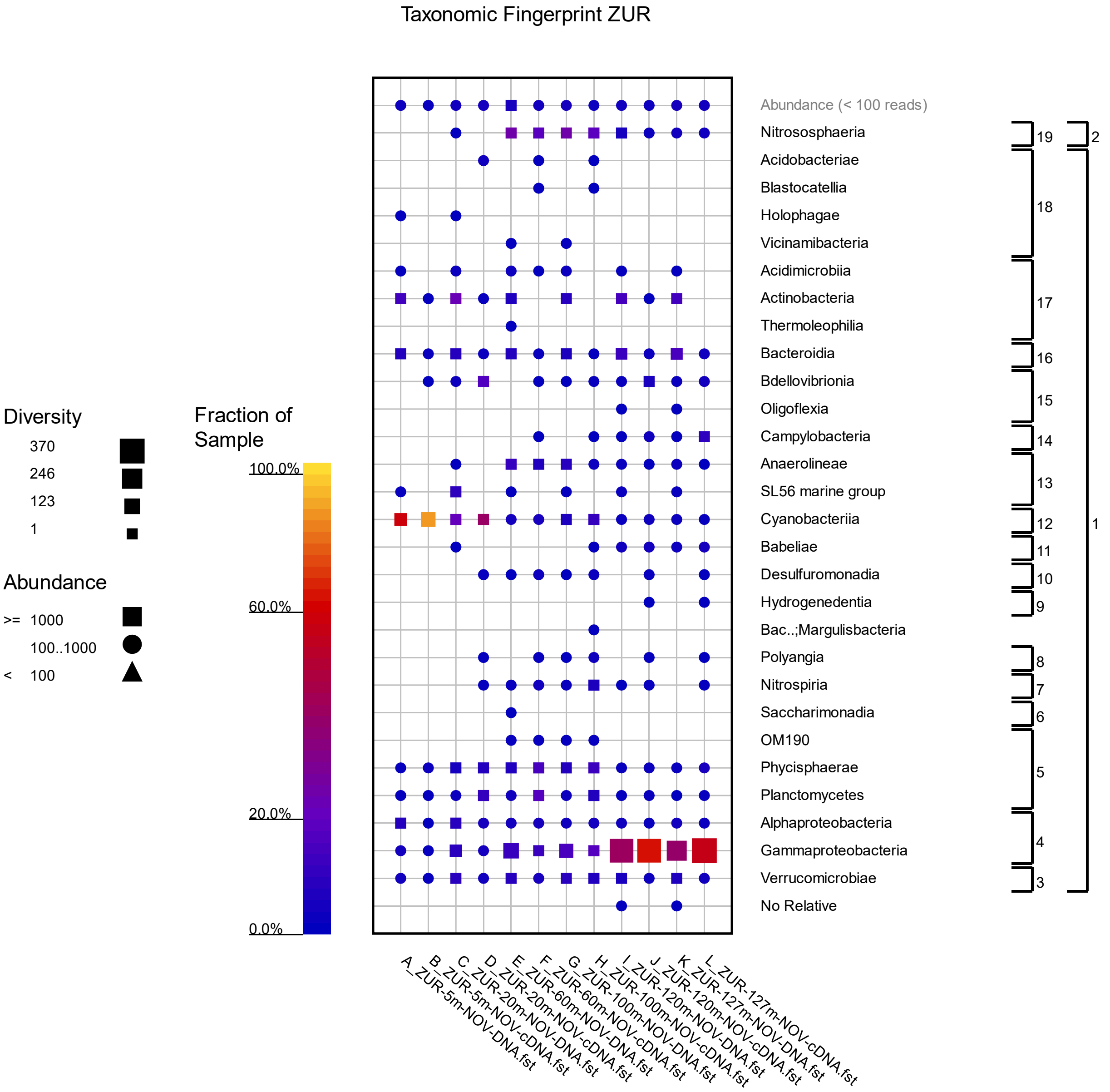

Supplementary Figure S1. Taxonomic fingerprint of the overall microbial diversity in lake ZUR. Taxonomic depth given is 3 (class), the diversity is also reflected by the size of the squares (# OTUs / path). Classes with reads <100 were excluded. These reads are displayed in the pseudo group "Abundance (< 100 reads)". The numbers behind the square brackets stand for the following domains and phyla: Bacteria (1), Archaea (2), Verrucomicrobiota (3), Proteobacteria (4), Planctomycetota (5), Patescibacteria (6), Nitrospirota (7), Myxococcota (8), Hydrogenedentes (9), Desulfobacterota (10), Dependentiae (11), Cyanobacteria (12), Chloroflexi (13), Campylobacterota (14), Bdellovibrionota (15), Bacteroidota (16), Actinobacteriota (17), Acidobacteriota (18), Crenarchaeota (19).

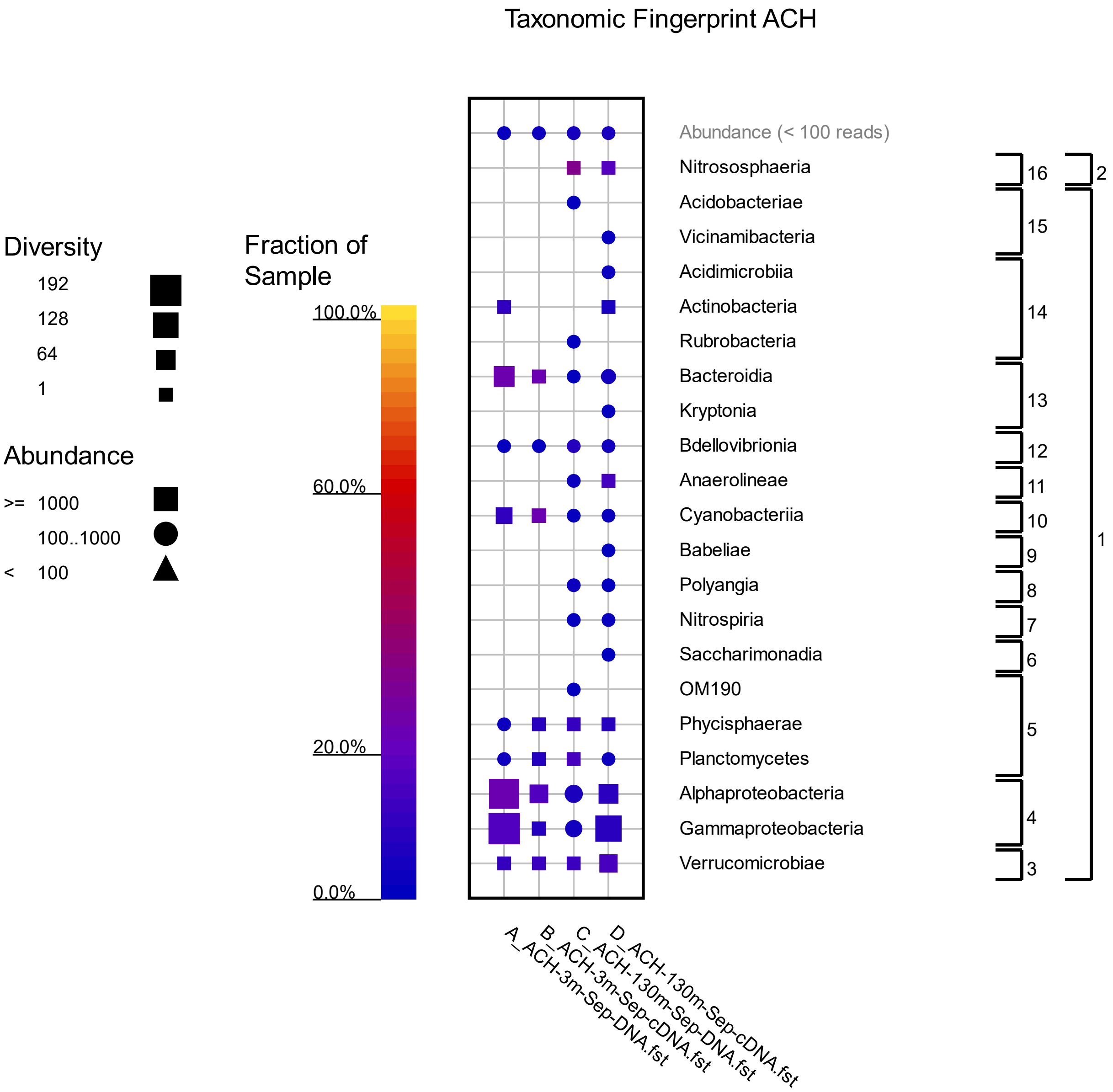

Supplementary Figure S2. Taxonomic fingerprint of the overall microbial diversity in lake ACH. Taxonomic depth given is 3 (class), the diversity is also reflected by the size of the squares (# OTUs / path). Classes with reads <100 were excluded. These reads are displayed in the pseudo group “Abundance (< 100 reads)”. The numbers behind the square brackets stand for the following domains and phyla: Bacteria (1), Archaea (2), Verrucomicrobiota (3), Proteobacteria (4), Planctomycetota (5), Patescibacteria (6), Nitrospirota (7), Myxococcota (8), Dependentiae (9), Cyanobacteria (10), Chloroflexi (11), Bdellovibrionota (12), Bacteroidota (13), Actinobacteriota (14), Acidobacteriota (15), Crenarchaeota (16).

Supplementary material Alfreider et al.

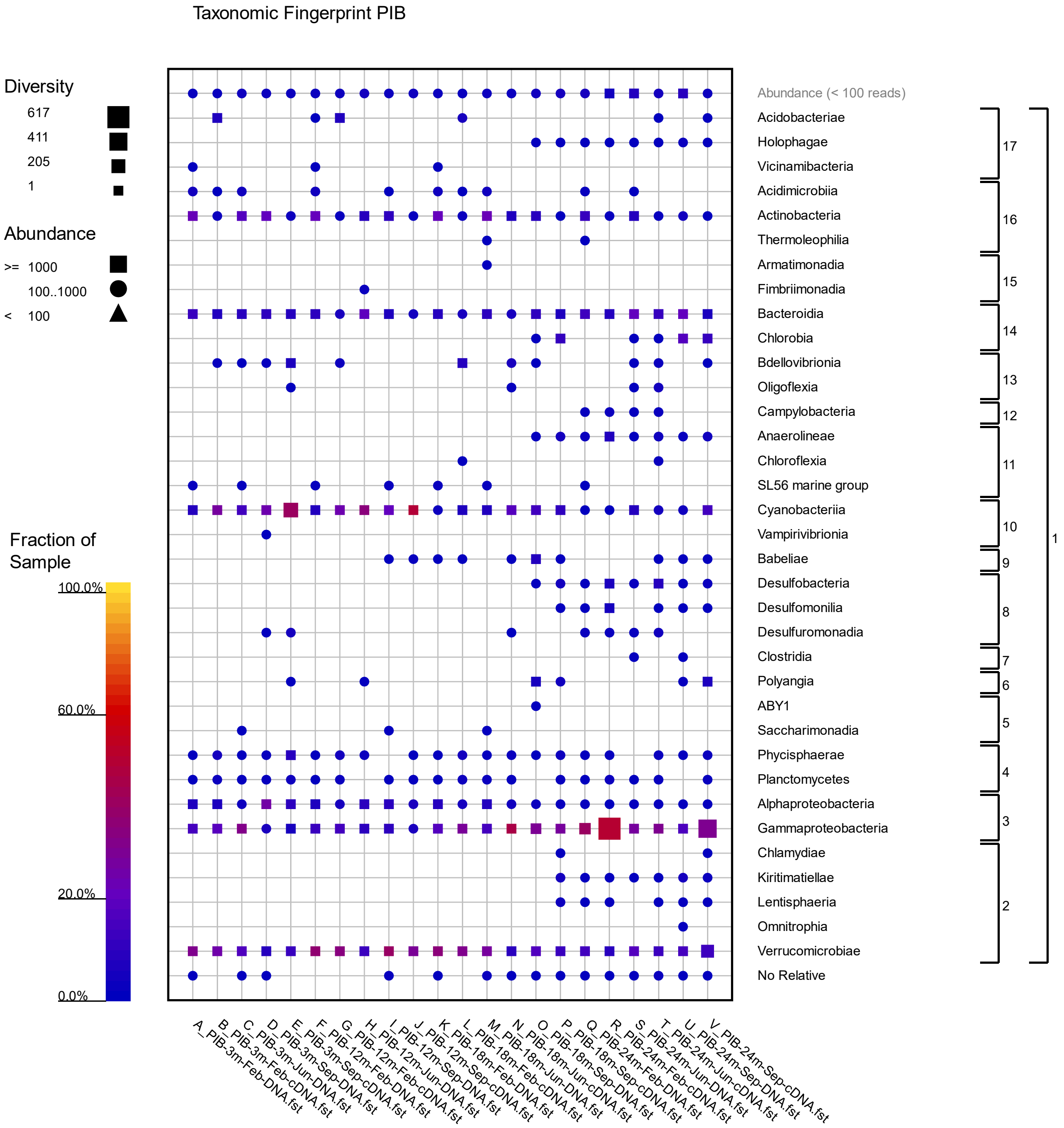

Supplementary Figure S3. Taxonomic fingerprint of the overall microbial diversity in lake PIB. Taxonomic depth given is 3 (class), the diversity is also reflected by the size of the squares (# OTUs / path). Classes with reads <100 were excluded. These reads are displayed in the pseudo group "Abundance (< 100 reads)". The numbers behind the square brackets stand for the following domains and phyla: Bacteria (1), Verrucomicrobiota (2), Proteobacteria (3), Planctomycetota (4), Patescibacteria (5), Myxococcota (6), Firmicutes (7), Desulfobacterota (8), Dependentiae (9), Cyanobacteria (10), Chloroflexi (11), Campylobacterota (12), Bdellovibrionota (13), Bacteroidota (14), Armatimonadota (15), Actinobacteriota (16), Acidobacteriota (17).

Supplementary material Alfreider et al.

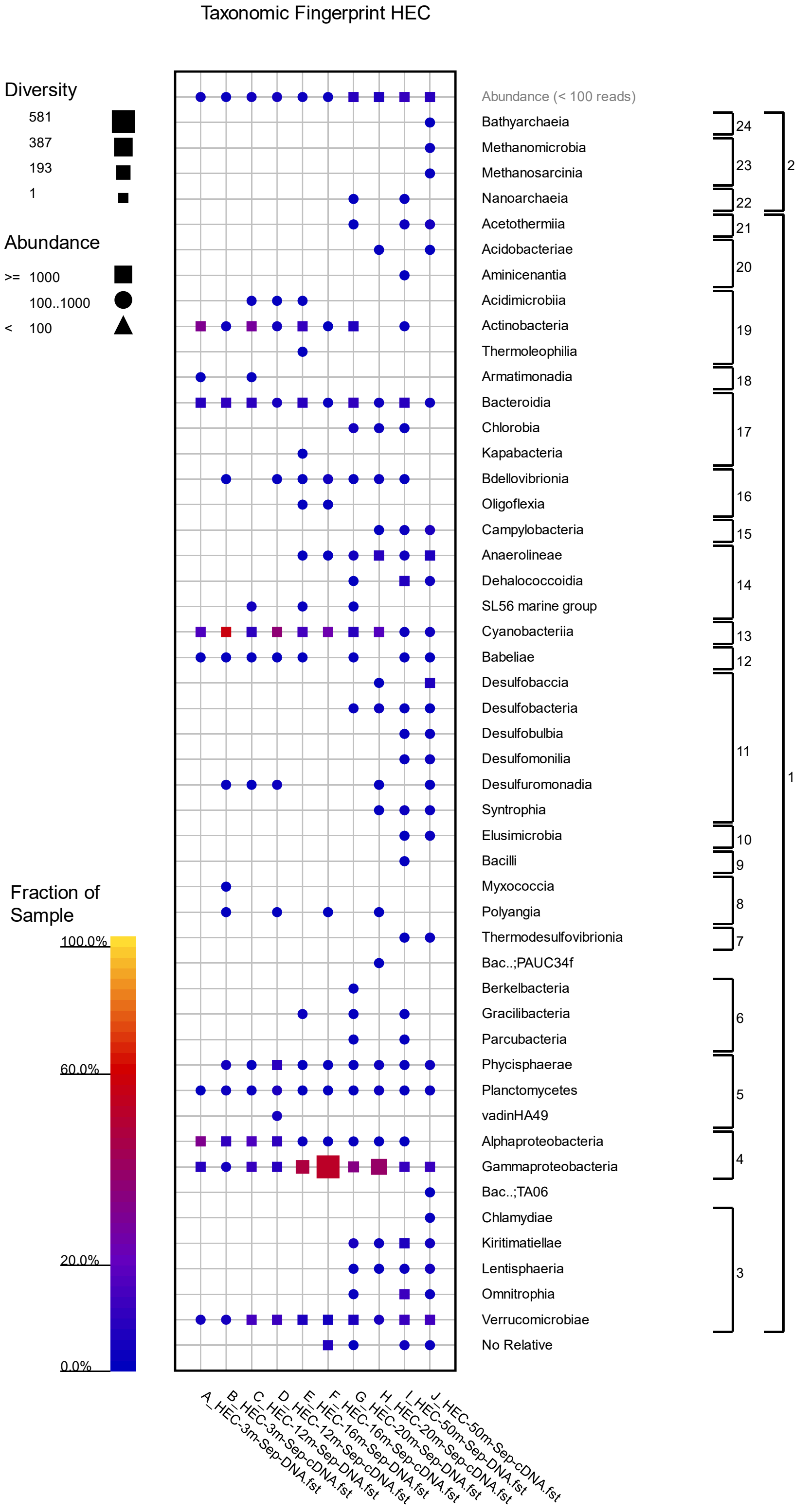

Supplementary Figure S4. Taxonomic fingerprint of the overall microbial diversity in lake HEC. Taxonomic depth given is 3 (class), the diversity is also reflected by the size of the squares (# OTUs / path). Classes with reads <100 were excluded. These reads are displayed in the pseudo group "Abundance (< 100 reads)". The numbers behind the square brackets stand for the following domains and phyla: Bacteria (1), Archaea (2), Verrucomicrobiota (3), Proteobacteria (4), Planctomycetota (5), Patescibacteria (6), Nitrospirota (7), Myxococcota (8), Firmicutes (9), Elusimicrobiota (10), Desulfobacterota (11), Dependentiae (12), Cyanobacteria (13), Chloroflexi (14), Campylobacterota (15), Bdellovibrionota (16), Bacteroidota (17), Armatimonadota (18), Actinobacteriota (19), Acidobacteriota (20), Acetothermia (21), Nanoarchaeota (22), Halobacterota (23), Crenarchaeota (24).

Supplementary Table S1. Physicochemical data used for the RDA analysis shown in Fig. 5.

Sampling Station	Temp. (°C)	Cond. (µS/cm)	Oxygen (%)	NO3-N (µg/L)	NH4-N (µg/L)	Ptot (µg/L)	DOC (µg/L)
PIB-3m-Feb	2,9	75,3	51	141	90	9,1	2036
PIB-3m-Jun	20,7	66,7	113	34	4	4,7	2567
PIB-3m-Sep	17,1	65,9	111	57	4	7,1	2451
PIB-12m-Feb	4	73,8	41	124	106	7,5	1976
PIB-12m-Sep	6,7	74,9	26	225	33	9,7	1990
PIB-18m-Feb	4	71	36	127	127	7,4	2028
PIB-18m-Jun	4,6	74,5	0,9	169	90	11,4	1977
PIB-18m-Sep	4,8	75,8	2,1	6	133	12,1	1926
PIB-24m-Feb	4,2	83,1	1,1	32	668	56,4	2270
PIB-24m-Jun	4,5	82,1	0	0	583	19,9	2371
PIB-24m-Sep	4,65	86,7	0	0	851	27,1	2328
HEC-3m-Sep	17,9	312	104	157	5	4,9	4111
HEC-12m-Sep	6,2	367	59	386	23	6,9	3316
HEC-16m-Sep	5,3	393	19	226	38	18,3	2987
HEC-20m-Sep	5,4	415	9	12	459	10,6	2737
HEC-50m-Sep	5,4	570	0	0	4564	185,7	2387

Supplementary Table S2. Spearman rank correlation comparing selected environmental parameters and *Ca*. Anammoximicrobium abundances derived by ddPCR analysis in the hypolimnion of lake PIB.

Variable	Depth	Temperature	Oxygen	NH_4-N
Ca. Anammoximicrobium	0.81*	-0.38	-0.81*	0.81*

Significant at: *p<0.01;
